# A Carbon Emission Measurement Method for Individual Travel Based on Transportation Big Data: The Case of Nanjing Metro

**DOI:** 10.3390/ijerph17165957

**Published:** 2020-08-17

**Authors:** Wei Yu, Tao Wang, Yujie Xiao, Jun Chen, Xingchen Yan

**Affiliations:** 1 College of Automobile and Traffic Engineering, Nanjing Forestry University, Longpan Road 159#, Nanjing 210037, China; yuweicar@163.com (W.Y.); xingchenyan.acad@gmail.com (X.Y.); 2 School of Architecture and Transportation, Guilin University of Electronic Technology, Jinji Road 1#, Guilin 541004, China; 3 School of Marketing and Logistics Management, Nanjing University of Finance & Economics, Wenyuan Road 3#, Nanjing 210046, China; yujiexiao@nufe.edu.cn; 4 School of Transportation, Southeast University, Dongnandaxue Road 2#, Jiangning Development Zone, Nanjing 210096, China; chenjun@seu.edu.cn

**Keywords:** carbon emission, carbon GSP, complex network, travel, metro, big data

## Abstract

With the strengthening of environmental awareness, the government pays much more attention to environmental protection and thus implements carbon trading schemes to promote the reduction of global carbon dioxide emissions. The carbon Generalized System of Preferences (GSP) is an incentive mechanism for citizens to value their energy conservation and carbon reduction. Individual travel needs to rely on various means of transportation, resulting in energy consumption. Carbon tax or subsidy can only be carried out after carbon GSP accurately measures individual carbon emissions. The big data acquired from the smart cards of passengers’ travels provide the possibility for carbon emission accounting of individual travel. This research proposes a carbon emission measurement of individual travel. Through establishing the network model of the Nanjing metro with a complex method, the shortest path of the passengers’ travels is obtained. Combined with the origination–destination (OD) records of the smart cards, the total distance of the passengers’ travels is obtained. By selecting the operation table to estimate the carbon emissions generated by the daily operation of the subway system, the carbon emissions per kilometer or per time of passenger travel are finally obtained. With the accurate tracking of carbon emissions for individual travel, the government may establish a comprehensive monitoring system so as to establish a carbon tax and carbon supplement mechanism for citizens.

## 1. Introduction

With the strengthening of environmental awareness, governments pay much more attention to environmental protection and are reducing their global carbon dioxide emissions. As an important supplement to the carbon trading scheme, the carbon Generalized System of Preferences (GSP) is an incentive mechanism for citizens and small (or micro) enterprises to value their energy conservation and carbon reduction. Carbon emission measurement for citizens becomes a critical problem for implementation of the carbon GSP.

Carbon tax is often targeted at enterprises or groups of a certain scale. The GSP is more suitable for evaluating individual carbon emissions. Carbon inclusion is the concrete performance of low-carbon rights and interests benefiting the public. Carbon GSP is an incentive mechanism established to endow citizens and small (or micro) enterprises with value in energy conservation and carbon reduction. With the accurate tracking of carbon emissions for passengers, the government may establish a comprehensive monitoring system so as to establish a carbon tax and carbon supplement mechanism for citizens and small (or micro) enterprises. Big data provide the possibility for such tracking. Various modes of transportation are also needed to be tracked, and the subway is an important part of it.

The main starting point of this research is the carbon GSP and the carbon emission tracking of individual public transport. The purpose of the GSP is to encourage residents to take public transport. Residents can get a certain amount of green travel points, so as to get compensation. Green points can be used as travel discounts, shopping subsidies, or personal tax breaks. Carbon GSP can increase residents’ awareness of environmental protection, encourage green travel, and promote residents to reduce future carbon emissions.

All kinds of transportation will produce carbon emissions. The government can distribute the share of carbon emissions to airlines, and passengers can get carbon compensation for it. Qi proposed a hybrid mechanism to assist governments in allocating carbon emission quotas to airlines and illustrated the practicability and effectiveness of this method in reducing carbon emissions through an example [1]. Zhang used the theory of communication to study whether information will affect the willingness of air passengers to accept carbon compensation. Airlines can design this information and improve it according to the past experiences of consumers [2]. Jiang proposed the evaluation system of technology progress of electric logistics vehicles, which can provide solutions for reducing carbon emissions of urban logistics [3].

Road car passenger transport will lead to a large amount of carbon emission, which needs to be controlled by different means. Zhang developed an empirical model to estimate the real fuel consumption and carbon dioxide emissions of gasoline-powered light buses in Macao [4]. Dhar used the energy system model to evaluate the emission reduction effect of Indian electric vehicles and analyzed the future Electric vehicle demand of India [5]. Peng established the cost-prediction curve of a marginal emission reduction of China’s passenger car industry in 2016–2030, as well as analyzed the impact of uncertainty factors on the cost of energy conservation and emission reduction [6].

From the perspective of the passenger transport industry, the carbon emission of urban passenger transport can be estimated. Ma proposed a carbon footprint ecological pressure model for the passenger transport industry and calculated the value of each province and autonomous region in China from 2006 to 2015 using the model [7]. Zhang used Shenzhen tourism survey data in 2014 to establish an intelligent model of passengers to study the future energy consumption and carbon dioxide emissions of urban passenger transport in 2014–2050 [8]. Wang analyzed the factors affecting carbon dioxide emissions in China’s passenger and freight sectors between 1990 and 2015. The results show that the population and transport structure promote the increase in transport carbon dioxide emissions [9].

Appropriate carbon tax policies can also reduce carbon emissions. Chen described an empirical study to measure the scale and variability of the carbon tax policy’s impact on carbon emissions in China’s provinces and believed that richer provinces need to levy higher carbon taxes [10]. Gupta used the simulation of system dynamics modeling to explore various situations related to fuel carbon tax and discussed whether carbon tax as a mitigation means can effectively reduce the carbon dioxide emissions of road passenger transport in India [11]. Dimitrovski used the original data collected by three different metropolises to analyze people’s willingness to pay in order to understand the effectiveness of carbon tax in India’s road passenger transport [12].

Urban development policies will affect greenhouse gas emissions. Adrian evaluated how the policy of reducing vehicle mileage affects the results of greenhouse gases and concludes that the choice is costly and inefficient [13]. Liu applied a structural equation model to study the impact of urban land-use characteristics on household travel and transportation energy consumption in Baltimore [14]. Song used a structural equation model to study the impact of the building environment on personal traffic emissions [15]. Pan proposed a social ecological simulation method to calculate and compare the greenhouse gas emissions of two cities [16]. Pan proposed a modeling method for greenhouse gas emissions caused by urban development and put forward suggestions for improvement [17].

Subways will produce carbon emissions in different parts of their life cycles, such as construction and operation, so different control measures need to be taken. Sanches evaluated the energy and carbon emission of a new metro line in Rio de Janeiro in its life cycle [18]. Lederer calculated the impact of three measures on reducing the green gas emission of the Vienna metro, and the results show that the effect of the metro is better than other modes of transportation [19]. Li used the life cycle assessment method to define the system boundary of the Shanghai metro life cycle, and made statistics of the relevant resource input and emission output according to the actual observation data. The results show that Shanghai metro has great energy saving potential [20].

To some extent, a subway will replace buses, so as to reduce carbon emissions. Wang has established four city-level emission models of passenger transport modes and customized the parameters, including power emission coefficient and fuel efficiency. The results show that the development of rail transit and further restrictions on cars can help to reduce carbon dioxide emissions [21]. Combined with quantitative and qualitative data collection and analysis, Andong believed that the low fuel efficiency of buses will lead to an increase in carbon dioxide emissions in the transport sector [22]. Soni analyzed the effect of the commuters’ shifts from other modes of transportation to subways on carbon dioxide reduction [23]. Liu allocated the total carbon emission of the city to different space areas according to the actual situation of Wuhan [24].

Optimizing the subway operation table and strengthening the environmental awareness in the construction process will reduce carbon emissions. Wang has established a time optimization model based on the train operation and passenger demand data, and the results show that the optimized time table can achieve better results in carbon emission and passenger carrying time [25]. Liu proposed a quantitative model of greenhouse gas emission based on a quota for subway station construction, which can improve the environmental awareness of the designers [26]. Liu studied the emission reduction potential of prefabricated subway station structures for greenhouse gases [27].

The complex network method has been applied in the network modeling of urban subway to analyze the evolution and various performances of the subway network. Zhang used the complex network method to establish the network model of three cities’ subway networks in China, as well as analyzed the network characteristics and robustness [28]. Yu analyzed the evolution of the Nanjing metro network by using a complex network method and combined it with the urban spatial pattern [29]. Wei established the supernetwork model of the Nanjing metro and analyzed its performance [30]. Yu analyzed the performance change of the Nanjing metro supernetwork by the simulation attack method [31]. Kanwar has carried out modeling and comparative analyses based on a complex network for the existing Delhi metro network and its expansion [32].

The existing economic strategies for carbon emission control include allocating carbon emissions to various transport companies or imposing a certain carbon tax. By calculating the fuel consumption of various means of transportation, we can also obtain the carbon emission of the transportation industry. From the perspective of a carbon footprint, carbon emissions can be measured from a macro perspective by using passenger transport data and the structure of the transportation system. The energy consumption of transportation facilities in the process of construction and operation can also be converted into carbon emissions, so it is necessary to strengthen the environmental awareness of all aspects of transportation. These methods are from the perspective of macro statistics to calculate carbon emissions.

Since China officially launched carbon emission trading in 2013, pilot work has been carried out in nine provinces or municipalities directly under the central government. At this stage, China’s carbon emissions trading has entered the stage of the carbon GSP, trying to more accurately track the carbon emissions of enterprises or individuals, so as to implement subsidies and other measures. The carbon GSP is a positive guidance mechanism that quantifies and endows a certain value to small enterprises, families, and individuals’ energy-saving and carbon reduction behaviors, and combines business incentives, policy incentives, and certified emission reduction transactions. The carbon dioxide equivalent is the unit of carbon emission reduction certified by the carbon GSP.

The calculation method of the carbon GSP is combined with the carbon footprint method. Based on the macro data of residents’ travels, it is assumed that a low-carbon public transport mode can replace the high-energy consumption transport mode, so as to calculate the carbon emission reduction. However, these methods cannot effectively track the carbon emissions caused by the individual travel of passengers. Only when the carbon emission is accurate to the individual level, can the passengers’ differences be distinguished effectively, so as to implement carbon emission trading or carbon compensation measures among residents. It is necessary to combine the macro emission data of the transportation industry with the passenger’s personal travel record, so as to accurately track the individual’s carbon footprint.

In 2017, the Guangdong Province of China made relevant regulations on the management and use of certified voluntary emission reductions generated by relevant enterprises or individuals involved in carbon GSP. The amount of carbon reduction generated by enterprises, families, and individuals can be converted into carbon emission through the carbon GSP platform. The carbon currency can be converted to preferential services in scenic spots, businesses, and other institutions.

The main low-carbon transportation modes in the city are new-energy buses and subways. Sharing bicycles and new-energy vehicles will also become an important part of travel. Nanjing metro can be familiar with the carbon market rules as a market participant, improve the ability of the urban transportation industry to adapt to the new situation of low-carbon development, and lay a foundation for the future transportation industry to fully integrate into the carbon emission trading system.

Due to the large number of small and micro emission sources, such as residents, as well as the emissions being small, single, and difficult to monitor, the calculation difficulty of a carbon reduction is far greater than that of large and medium-sized industrial enterprises. However, big traffic data offer the possibility to track the exact emissions of individual passengers. This research attempts to accurately estimate the carbon emissions of passengers caused by taking the subway. The distance model-based complex network can calculate the exact distance of each passenger’s daily OD (origination–destination) travel by analyzing the data from the passengers’ smart cards. At the same time, data can be obtained and calculated through the operation table of the subway to estimate the carbon emissions generated by subway, to finally get the carbon emissions of the passengers per kilometer. The process and method can effectively calculate the carbon emissions of the different travel distances of passengers, thus providing some quantitative methods for the implementation of the carbon GSP in China.

## 2. Analysis Process

### 2.1. Situation of the Nanjing Metro Network

Nanjing metro adopts an automatic ticket checking system, which accumulates big traffic data through the smart card of passengers and can be used to analyze the travel rules of passengers. Yang investigated commuters using public bicycles entering the subway, and analyzed their personal characteristics and experiences before and after going to work [33]. Li studied the influence of weather conditions on the single line passenger flow of Nanjing metro [34]. Zhao used the data of the Nanjing bus smart card to analyze the transfer between a subway and bus [35]. Wei used the smart card data of Nanjing metro to analyze the temporal and spatial changes of passenger flow [36]. Wei used the smart card data to identify the abnormal behavior of passengers, and classified the abnormal behavior [37].

The Nanjing metro lines infrastructure data include the line name, opening times, number of stations, and length. Seven metro lines had been opened. Table 1 shows the Lines, and Line 1, 2, 3, 4 are the main lines.

**Figure 1:**
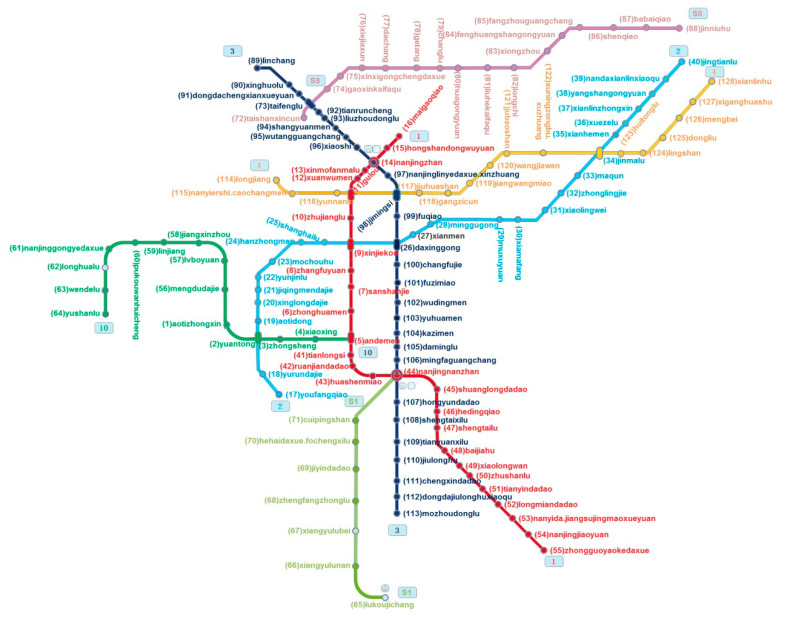
Nanjing metro map.

Figure 1 shows the Nanjing metro map in 2017 with different colors representing different lines and stations. The station number indicates the code in the Nanjing metro management system.

### 2.2. Problem Description

The research problem can be summarized as follows: How does one track the individual carbon emission of subway passengers? We need to consider the overall emissions of the subway and the specific travel of the passengers. The overall emission of a subway is affected by the network structure, line operation, vehicle type, and other factors. The passenger’s specific travel includes the time and stations of entry and exit, which are recorded by the passenger’s smart card.

By analyzing the swiping card records of the passengers, we can suppose the travel choice of a passenger, and then calculate the travel distance of the passenger. Taking a certain period of time as the statistical interval, the average carbon emissions of the travel can be obtained by using the total emissions of the metro lines and divide that by the number of all the travels or the total distance of all the passengers.

Finally, combined with the passenger’s specific travel, the carbon emissions of each passenger can be tracked to meet the needs of the calculating carbon subsidy. This method is not only suitable for a subway network, but also for buses, public bicycles, and other public transport modes.

### 2.3. Analysis Process of Individual Carbon Emission

**Figure 2:**
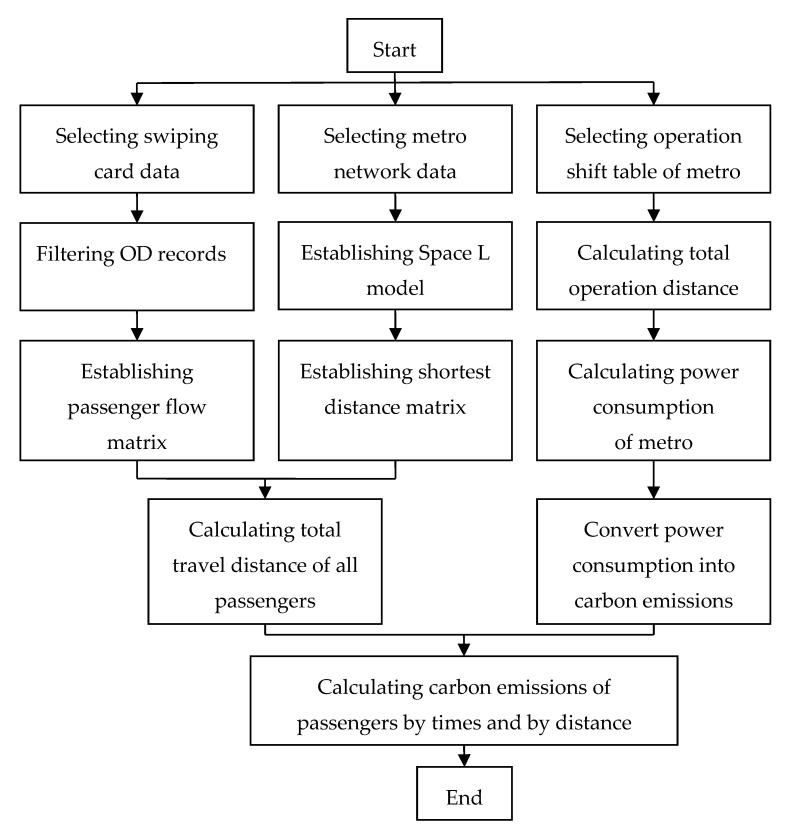
Analysis flow chart.

Figure 2 shows the analysis flow chart.

The analysis process shows how the individual carbon emission is calculated by the number trips or distance.

(1) Step 1: Select the swiping card data of Nanjing metro passengers in some days and filter out the abnormal OD records. The filtering process of the smart card data was proposed by the literature [37].

Only after these abnormal records are filtered can we get the reasonable travel records of the passengers. Even within 300 min, there may be multiple round trips for passengers staying between stations for a long time. This part of the data was not much; it was treated as normal data.

(2) Step 2: Establish the passenger flow matrix by day.

The corresponding flow adjacency matrix $F={\left\{f_{ij}\right\}}_{N\times N}$ can be defined as Formula (1).

(1) $$f_{ij}=\{\begin{array}{ll} w_{ij}, & i\neq j\\ 0, & i=j \end{array}$$

where $w_{ij}$ is the weight of the edge, representing the flow from node$v_{i}$ to node$v_{j}$.

(3) Step 3: Establish the Space L model of the Nanjing metro network and the shortest distance matrix between the metro stations.

The Space L model was established according to the connection relationship between two adjacent stations on the same subway line.

The corresponding adjacency matrix $M={\left\{m_{ij}\right\}}_{N\times N}$ of the metro network can be defined as Formula (2).

(2) $$m_{ij}=\{\begin{array}{ll} 1, & (v_{i},v_{j})\in l_{k}\\ 0, & (v_{i},v_{j})\notin l_{k} \end{array}$$

where $m_{ij}$ is the relationship between two adjacent stations, namely node$v_{i}$ and node$v_{j}$. $l_{k}(1\leq k\leq K)$ replaces the metro lines, and K is the number of metro lines.

According to the metro network, the shortest distance network $S={\left\{s_{ij}\right\}}_{N\times N}$ can be deduced. The shortest distance reflects the length of the shortest passing through any two nodes. The corresponding adjacency matrix can be defined as Formula (3).

(3) $$s_{ij}=\{\begin{array}{ll} {\min(d}_{ij}), & i\neq j\\ 0, & i=j \end{array}$$

where $d_{ij}$ is the distance from node $v_{i}$ to node $v_{j}$ of the metro network *M*.

(4) Step 4: Calculate the total travel length of passenger flow according to the passenger flow matrix and the shortest distance matrix.

The length of OD travel of all passengers can be defined as Formula (4).

(4) $$L_{f}=\sum_{i=1}^{N}\sum_{j=1}^{N}f_{ij}s_{ij}$$

where $L_{f}$ is the length of the OD travel of all passengers, $f_{ij}$ is the passenger flow from node $v_{i}$ to node $v_{j}$, $s_{ij}$ is the shortest distance from node $v_{i}$ to node $v_{j}$,and N is the number of metro stations.

(5) Step 5: Select the operation shift of the metro lines in some days, and calculate the total operation distance of the metro lines by day. According to metro vehicle type and the total running distance, calculate the power consumption of the metro and convert it into carbon emissions.

The following parameters refer to the values in a certain day. The carbon emission of all metro lines can be defined as Formula (5).

(5) $$C=R\sum_{k=1}^{K}L_{k}E_{k}F_{k}$$

where C is the carbon emission of all the metro lines,R is the conversion rate of energy consumption to carbon emission, $L_{k}$ is the length of the metro line i,$E_{k}$ is the energy consumption per kilometer of the septic type of metro vehicle, $F_{k}$ is number of runs of the metro line i, and K is the number of the metro lines.

(6) Step 6: According to the carbon emissions of the subway network and the total travel length of the passenger flow, calculate the carbon emissions of the passengers by times and by distance.

The carbon emission per capita per time can be defined as Formula (6).

(6) $$C_{pt}=C/(\sum_{i=1}^{N}\sum_{j=1}^{N}f_{ij})$$

where $C_{pt}$ is the carbon emission per capita per time, C is the carbon emission of all the metro lines, $f_{ij}$ is the passenger flow from node $v_{i}$ to node $v_{j}$, and N is the number of metro stations.

The carbon emission per capita per kilometer can be defined as Formula (7).

(7) $$C_{pk}=C/L_{f}$$

where $C_{pk}$ is the carbon emission per capita per kilometer, C is the carbon emission of all the metro lines, and $L_{f}$ is the length of the OD travel of all passengers.

According to the number of trips or the distance of an individual’s travel over a specific period, the carbon emission of an individual can be calculated. If the average carbon emission value of all the travel is calculated according to the total emissions of the metro lines and passenger flow, and the different distances of the residents’ travels are ignored, the results calculated by distance are more accurate than by times.

## 3. Data Filtering and Establishing the Model

### 3.1. Data Filtering of the Nanjing Metro

By the end of 2016, Nanjing had 6.78 million urban residents. The residents choose the metro as the main mode of public transportation. Nanjing Metro Group uses an automatic fare collection system to manage the passengers’ travels. Passengers can use long-term smart cards for public transportation or reusable temporary smart cards. The passenger flow data includes card number, card type, inbound and outbound station, arrival and departure times, etc. Table 2 shows the data filtering of the OD cards in a week.

The abnormal data include the records of inbound before yesterday, inbound after today, negative travel time, beyond 300 min, from 0 to 1 min, and inbound and outbound on the same stations [32]. The first two kinds of records are caused by statistical errors. Negative travel time is caused by device running error. The Nanjing metro management department has stipulated that passengers cannot stay in the subway network for more than 300 min. The 0–1 min record is because the passengers did not actually take the metro. The records of inbound and outbound on the same stations are not necessary to be analyzed.

The behavior of in and out of the same station was treated as a kind of abnormal data point during the data filtering. Such behavior in the 0–1 min interval may be to evade tickets or using the site as a temporary channel. Another kind of behavior in the normal interval of 1–300 min in the same station may be marketing behavior in the subway. Because the existing card data cannot track the second type of behavior in distance, this kind of data is filtered. In a future study, if the location data of mobile phones can be obtained, one may be able to identify such behavior the same station in distance.

**Figure 3:**
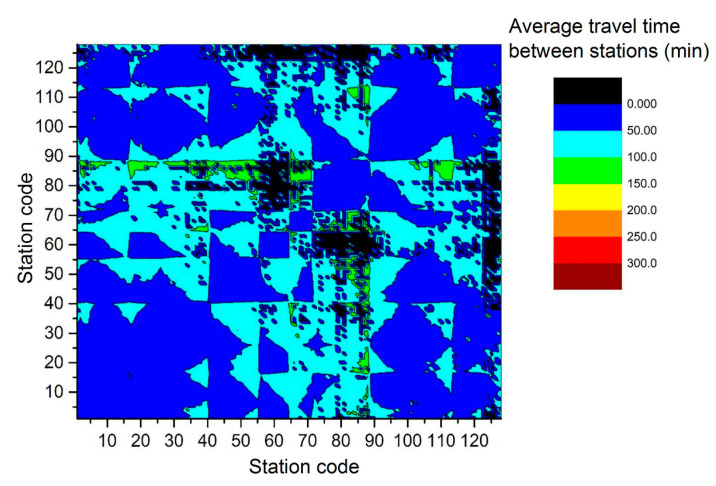
Average time between stations on 13 February 2017.

Figure 3 shows the thermograph of the average time distribution between stations on 13 February 2017. Average time refers to the average OD travel time between the different stations in a day. The distribution of the remaining six days is similar to this one. Most data are in the 0–100 min range. Some data are zero. Few data exceed 150 min.

**Figure 4:**
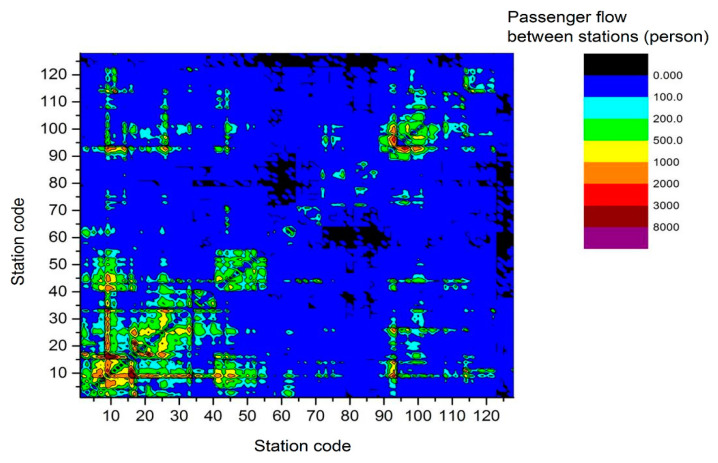
Passenger flow distribution between stations on 13 February 2017.

Figure 4 shows the thermograph of the distribution of passenger flow between stations on 13 February 2017. Part of the passenger flow between the stations is zero, which is caused by the inconvenient communication between the remote stations and other stations. Most of the passenger flow is 0–100 persons, and some are 100–200 persons and 200–500 persons. There are also some passenger flows with more than 2000 persons. Line 1, 2, and 3 are the main lines, and the passenger flow on the same line or between these lines is large.

### 3.2. Establishing the Complex Network Model

It is assumed that the route of the passenger travel is based on the selection of the shortest distance. The complex network method is helpful for the visualization analysis of the subway lines [26]. Generally, Space L and Space P models can be used to establish a subway network model with a complex network. The Space L model can reflect the distance relationship of the stations in space. The Space P model can reflect the times of transfer between the stations. This research assumes that the passengers’ travel between any two stations is aimed at the shortest distance, which is more suitable for modeling with the Space L model.

**Figure 5:**
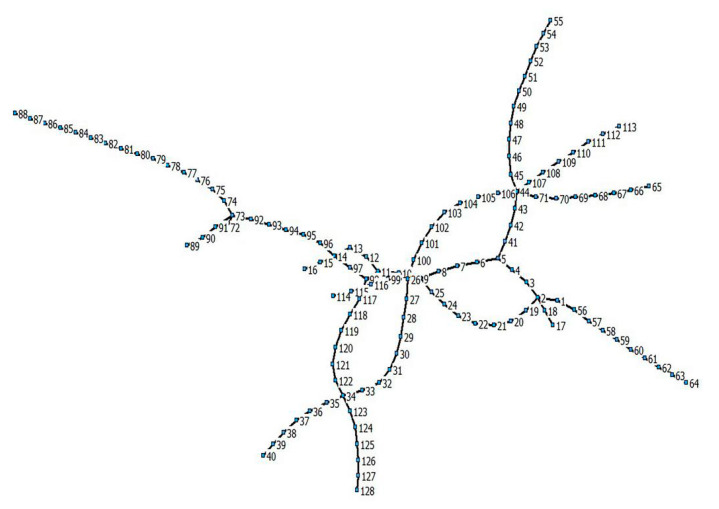
Space L model of the Nanjing metro network.

The Space L model uses the adjacent subway stations to connect, which reflects the relationship between the stations in the actual geographical space. Figure 4 shows the Space L model of the Nanjing metro network. The figure was drawn by Netdraw software (Analytic Technologies, Lexington, KY, USA). Figure 1 and Figure 5 show a clear corresponding relationship in the Nanjing metro network, depicting the regional structure and transportation hub of Nanjing. The network node number is consistent with the subway station code.

## 4. Results

### 4.1. Running Mileage of the Nanjing Metro Lines

**Figure 6:**
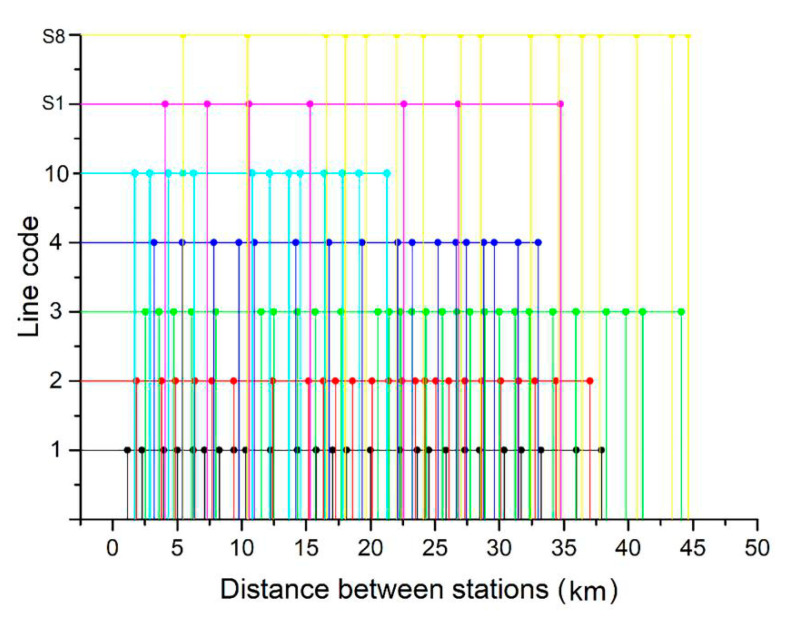
Distance between stations of the Nanjing metro lines.

Figure 6 shows the distances between stations of the Nanjing metro lines, reflecting the distances between stations and the line length.

Table 3 shows the operation schedule of the Nanjing metro, including the line code, up direction and time, and down direction and time. Train operation routing is one of the main technical standards of rail transit lines. It is based on the peak hour section passenger flow predicted in each design year, combined with the feasibility and economy of the project, to determine the number of train formations in each operation section, the turn back station, and the train operation pairs in each operation section and peak hour. The number in up direction and down direction replaces the code of the origination and destination stations. A reasonable train operation routing can not only meet the service level, but also improve the efficiency of the train operation, avoid a waste in transport capacity, and make the operation organization economic and reasonable.

The Nanjing rail transit train adopts the mode of a large and small routing operation. According to the needs of the passenger flow, trains with different marshalling and operation pairs can be organized to operate in each section to meet the needs of passenger flow and improve the operation efficiency. Table 4 shows the running mileage of the Nanjing metro lines, including the line code, large routing, and small routing. The large routing is in full operation, while the small routing is in sectional operation.

### 4.2. Vehicle Type and Energy Consumption of Nanjing Metro

Table 5 shows the performance of the Nanjing metro trains. Nanjing metro has selected two models, namely A and B2.

Nanjing metro vehicles use electricity as the driving energy. Although electricity is a kind of clean energy, considering its source, it will also produce carbon dioxide emissions. There is no standard for electricity and carbon dioxide production. This research adopts the conversion method of electric energy and standard coal. The State Energy Administration of China publishes a statistical report on the national power industry every year. According to the report in 2017, the standard coal consumption of power plants with the power consumption of 6000 kW and above is 0.000308 tce. The conversion value of standard coal and carbon dioxide recommended by the Energy Research Institute of China’s National Development and Reform Commission is 2456.7 kg/tce. The two parameters can be multiplied to obtain the conversion value of the kilowatt hour and carbon dioxide in 2017, which was 0.757 kg/kwh.

Table 6 shows the vehicle types and energy consumption of the Nanjing metro lines per day. The total energy consumption and carbon emission of each line can be calculated according to the vehicle type, energy consumption, and running mileage of the different lines. Since the daily operation schedule of the subway in the selected date remains unchanged, the carbon emission of the line also remains stable. The total energy consumption per day of the seven lines is 263,372.236 kwh, and the carbon emission is 199,373.783 kg.

### 4.3. Carbon Emission of Passengers by Times and Distance

Table 7 shows the carbon emission per capita in a week, which includes two sets of data calculated by travel times and travel distance per day. The daily OD records are between 1 million and 1.3 million, and the per capita distance is about 12 km. The per capita emission by distance is between 0.012 and 0.014 kg/km, and the per capita carbon emission by times is between 0.152 and 0.185 kg. The fluctuation range of the whole numerical value is not large, and it keeps a stable trend. After getting the carbon emission per capita by distance and tracking the specific total personal travel distance, we can accurately calculate the carbon emission per day of personal travel by subway.

It can be seen from the results that the carbon emission value per capita per kilometer is more stable than that of per capita per time. This is because the former considers the different distances of all passengers, while the latter is only related to passenger flow. For different passengers, if the carbon emissions are calculated according to travel distance, it can truly reflect the difference in travel routes. If the carbon emission is calculated according to the number of trips, it cannot reflect the differences in passenger travel and cannot effectively evaluate the carbon emission.

## 5. Discussion

The government began to attach importance to environmental protection to ensure the improvement of the living quality of residents. Guided by the government, carbon trading between enterprises and residents is an effective means of environmental control. The carbon GSP is suitable for energy conservation and carbon reduction of citizens and small (or micro) enterprises. Various behaviors and activities of enterprises and residents can be converted into carbon emissions by the carbon GSP, so as to implement carbon compensation measures. However, as a small emission source, residents have the problem of small emissions, which is difficult to trace and calculate.

This research can accurately calculate the carbon emissions of individual travel of residents by transportation big data. Improving transportation big data and the implementation of the carbon GSP would need the government to further guide and establish a comprehensive monitoring system, so as to establish a carbon supplement mechanism suitable for individuals or enterprises.

## 6. Conclusions

Transportation is one of the important sources of carbon emissions. Rail transit, various new energy and sharing concepts have brought new opportunities for carbon emission reduction. Many cities have opened subway lines as a part of their comprehensive transportation systems. The convenience of subways also urges residents to choose the subway as the main mode of transportation.

The purpose of this research is to accurately calculate the carbon emissions of residents traveling by subway. The wide application of smart cards in transportation system provides the possibility of accounting for tracking the individual carbon emissions of residents. The automatic ticket checking system used by the Nanjing metro accumulates the big data of the residents’ travel.

The complex network model of the Nanjing metro was established to get the shortest path between the different stations. At the same time, the operation table of the subway was selected to estimate the carbon emissions of the daily operation of the subway. Finally, the carbon emissions per kilometer of passenger travel were obtained. For passengers, according to the total distance they travel every day, they accrue carbon emissions by taking the subway every day. The method and the case studied here can accurately calculate the individual emissions of subway passengers, thus laying the foundation for the implementation of the carbon GSP.

There are still some shortcomings in this research method. The operation schedule of the subway will change frequently, and it will be different on weekends and holidays. It needs to be tracked for a long time. The carbon emission of metro vehicles is not a fixed value, but changes with the working conditions. It is necessary to record the situation of each metro operation. More accurate models are needed for passenger route assignment and estimation to reflect individual differences. The subway needs to be monitored in combination with other modes of transportation, using smart card data for more extensive tracking.

## Figures and Tables

**Table 1:** Situation of the Nanjing metro lines.

Opening Sequence	Number of Stations	Opening Year
1	27	2005
2	26	2010
10	14	2014
S1	8	2014
S8	17	2014
3	29	2015
4	18	2017

**Table 2:** Data filtering of the origination–destination (OD) records in a week.

Date	Week	Whole Number	Number after Filtering	Inbound before Yesterday	Inbound after Today	Negative Travel Time	>300 min	0–1 min	Inbound and Outbound on the Same Station
2.13	Monday	1,228,131	1,218,435	93	29	21	1240	1569	6744
2.14	Tuesday	1,305,521	1,294,958	176	44	14	1316	1650	7363
2.15	Wednesday	1,240,308	1,229,713	248	25	18	1296	1743	7265
2.16	Thursday	1,201,713	1,192,098	68	28	22	1312	1646	6539
2.17	Friday	1,324,342	1,313,355	72	28	11	1313	1719	7844
2.18	Saturday	1,190,690	1,180,916	63	25	14	1170	1275	7227
2.19	Sunday	1,089,382	1,079,395	416	24	5	1118	1245	7170

**Table 3:** Operation schedule of the Nanjing metro.

Line	Up Direction	Up Time	Down Direction	Down Time
1	55–16	5:47–23:27	16–55	5:42–23:19
2	17–40	6:00–23:00	40–17	6:00–23:00
3	89–113	6:00–23:00	113–89	6:00–23:00
4	114–128	6:00–23:00	128–114	6:00–23:00
10	5–64	6:00–23:40	64–5	5:40–23:00
S1	65–44	6:00–22:40	44–65	6:00–22:40
S8	88–72	6:00–22:00	72–88	6:00–22:00

**Table 4:** Running mileage of the Nanjing metro lines.

Line	Large Routing				Small Routing				Total Running Mileage (km)
	Direction	Number	Round Trip Time (min)	Length (km)	Direction	Number	Round Trip Time (min)	Length (km)	
1	55–16	314	133	37.918	46–16	240	91	23.607	17,571.93
2	17–40	368	126	37.009	33–40	20	36	12.394	13,867.19
3	89–113	212	159	44.104	89–108	160	129	34.148	14,813.73
4	114–128	210	107	31.443	114–125	148	85	25.166	10,327.60
10	5–64	316	78	21.261					6718.476
S1	65–44	225	80	34.737					7815.825
S8	88–72	206	116	44.619					9191.514

**Table 5:** Performance of the Nanjing metro vehicles.

Performance	Vehicle Type	
	A	B2
Width (m)	3	2.89
Length of the motor car (m)	22.8	19
Number of motors	4	4
Number of trailer formations	2	2
Capacity (person)	1860	1820
Power of traction motor (kw)	200	190
Power of train (kw)	3200	3040
Maximum speed (km/h)	80	100
Energy consumption per kilometer (kwh/km)	3.122	3.585

**Table 6:** Vehicle types and energy consumption of the Nanjing metro lines per day.

Line	Vehicle Type	Energy Consumption per Kilometer (kwh/km)	Running Mileage (km)	Total Energy Consumption (kwh)	Carbon Dioxide Emission (kg)
1	A	3.122	17,571.932	54,859.572	41,528.696
2	A	3.122	13,867.192	43,293.373	32,773.083
3	A	3.122	14,813.728	46,248.459	35,010.083
4	B2	3.585	10,327.598	37,024.439	28,027.500
10	A	3.122	6718.476	20,975.082	15,878.137
S1	B2	3.585	7815.825	28,019.733	21,210.938
S8	B2	3.585	9191.514	32,951.578	24,944.345

**Table 7:** Carbon emission per capita in a week.

Date	Running Distance of All Passengers (km)	Number of OD Records	Distance per Capita (km)	Carbon Dioxide Emission per Capita per Kilometer (kg/km)	Carbon Dioxide Emission per Capita per Time (kg)
2.13	14,699,169.280	1,218,435	12.064	0.014	0.164
2.14	15,293,766.580	1,294,958	11.810	0.013	0.154
2.15	14,809,239.060	1,229,713	12.043	0.014	0.162
2.16	14,294,860.670	1,192,098	11.991	0.014	0.168
2.17	16,007,539.650	1,313,355	12.188	0.012	0.152
2.18	14,870,032.230	1,180,916	12.592	0.014	0.169
2.19	13,918,467.500	1,079,395	12.895	0.014	0.185
